# Caffeic Acid Attenuates Multi-Drug Resistance in Cancer Cells by Inhibiting Efflux Function of Human P-Glycoprotein

**DOI:** 10.3390/molecules25020247

**Published:** 2020-01-07

**Authors:** Yu-Ning Teng, Charles C.N. Wang, Wei-Chieh Liao, Yu-Hsuan Lan, Chin-Chuan Hung

**Affiliations:** 1 Department of Medicine, College of Medicine, I-Shou University, 8 Yida Road, Kaohsiung 82445, Taiwan; eunicegh520@gmail.com; 2 Department of Bioinformatics and Medical Engineering, Asia University, 500 Lioufeng Rd., Wufeng, Taichung 41354, Taiwan; chaoneng.wang@gmail.com; 3 Department of Pharmacy, College of Pharmacy, China Medical University, 91 Hsueh-Shih Road, Taichung 40402, Taiwan; u102003316@cmu.edu.tw; 4 Department of Pharmacy, China Medical University Hospital, 2 Yude Road, Taichung 40447, Taiwan

**Keywords:** caffeic acid, cancer multidrug resistance, P-glycoprotein, phenolic acid

## Abstract

Multidrug resistance (MDR) is a complicated ever-changing problem in cancer treatment, and P-glycoprotein (P-gp), a drug efflux pump, is regarded as the major cause. In the way of developing P-gp inhibitors, natural products such as phenolic acids have gotten a lot of attention recently. The aim of the present study was to investigate the modulating effects and mechanisms of caffeic acid on human P-gp, as well as the attenuating ability on cancer MDR. Calcein-AM, rhodamine123, and doxorubicin were used to analyze the interaction between caffeic acid and P-gp, and the ATPase activity of P-gp was evaluated as well. Resistance reversing effects were revealed by SRB and cell cycle assay. The results indicated that caffeic acid uncompetitively inhibited rhodamine123 efflux and competitively inhibited doxorubicin efflux. In terms of P-gp ATPase activity, caffeic acid exhibited stimulation in both basal and verapamil-stimulated activity. The combination of chemo drugs and caffeic acid resulted in decreased IC_50_ in *ABCB1*/Flp-In^TM^-293 and KB/VIN, indicating that the resistance was reversed. Results of molecular docking suggested that caffeic acid bound to P-gp through GLU74 and TRY117 residues. The present study demonstrated that caffeic acid is a promising candidate for P-gp inhibition and cancer MDR attenuation.

## 1. Introduction

As cancer is one of the leading causes of death worldwide, cancer treatment is always on the top of listed hot research topics [1]. With advanced scientific researches and abundant medical resources in recent decades, diverse options have been developed to conquer cancer and related diseases. Nevertheless, the multi-drug resistance (MDR) of cancer treatment is still an ever-changing problem and more in-depth studies have been conducted to unveil the complicated characteristics of cancer treatment. Cancer MDR manifests cross resistance to several structurally and mechanically different chemo-agents and could be contributed to the following reasons [2]. The change in tumor microenvironment [3,4,5], decreased drug uptake [6], adapted cell apoptotic pathways [7,8,9], drug inactivation through metabolism [10,11], the influence of epigenetic regulation [12,13], mutation of drug target site [14], and the increased drug efflux [15] have been reported to play important roles in causing cancer MDR. Among the above mechanisms, the increased drug efflux by ATP-binding cassette (ABC) transporters has been regarded as the most influential cause. ABC transporter superfamily consists of several subfamilies, and P-glycoprotein (P-gp) is one of the most comprehensively studied proteins [16]. P-gp is encoded by human *ABCB1* gene and can recognize various clinically used drugs, including antidepressants, HIV protease inhibitors, immunosuppressive agents, and chemotherapeutic drugs [17,18]. The diverse structures recognizing and effluxing the ability of P-gp, result in insufficient chemo-drug concentration inside cancer cells, therefore, causing cancer MDR.

There have been a series of P-gp inhibitor developments along the cancer MDR reversing agents discovering history, and the improvements have been based on previous failure experiences [19]. First generation P-gp inhibitors are potent but toxic as the required dose is high; examples of this are quinidine and verapamil [20]. Second generation inhibitors have exhibited better effects with lower IC_50_, but the involvement of these inhibitors in CYP450 interaction has impeded their further application [21,22]. Third generation inhibitors, including tariquidar and zosuquidar, have demonstrated prominent MDR reversal effects. However, they have still faced failure in clinical studies [23,24]. Therefore, severe toxicity and interaction of the above chemical reagents have turned the research direction toward natural resources, aiming at discovering low toxic and potent structures from plants, fungi, or marine organisms.

**Figure 1:**
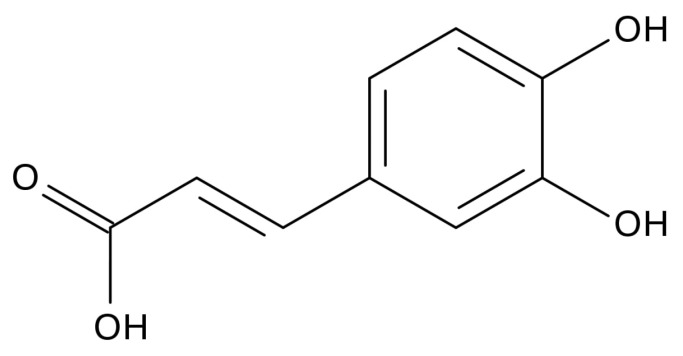
The chemical structure of caffeic acid.

Among various natural resources, phytochemicals such as flavonoids and phenolic acids get much attention due to their multiple pharmacological effects, including antioxidant and antitumor activity [25,26]. Several phytochemicals, such as cyanidin, catechin, quercetin, caffeic acid, and ellagic acid, have been related to the down-regulation of human LDL oxidation [27]. Ellagic acid and ursolic acid have been reported to exhibit preventive and therapeutic effects against breast cancer cells [28]. Caffeic acid (Figure 1), a phenolic acid that widely exists in vegetables, fruits, and tea extracts, is well-known as a natural antioxidant [29]. Besides, caffeic acid has also been identified to have anti-inflammatory, antibacterial, and antiviral effects [30,31]. With regards to cancer treatment, caffeic acid and its derivative, caffeic acid phenethyl ester (CAPE), exhibit some therapeutic effects toward lung cancer and breast cancer cells, as well as breast cancer pre-clinical models [32,33,34]. CAPE has been well studied in previous researches, including its *MDR1* gene down-regulating effects in MCF-7 and MDA-MB-231 breast cancer cells [34] and P-gp inhibitory effects in HeLa resistant cancer subline and human intestinal LS174T cell line [35,36]. Nevertheless, the P-gp inhibitory and MDR modulating information of the caffeic acid was insufficient and warrants further detailed investigation.

Therefore, in the present study, comprehensive researches of caffeic acid were conducted. The interaction of caffeic acid with human P-gp, as well as the inhibitory effects and mechanisms were assessed in P-gp over-expressing cell line *ABCB1*/Flp-In^TM^-293. The cancer MDR reversing ability of caffeic acid was then evaluated in both *ABCB1*/Flp-In^TM^-293 and KB/VIN MDR cancer cell lines. The present study demonstrated that caffeic acid is a promising candidate for P-gp inhibition and cancer MDR attenuation.

## 2. Results

### 2.1. Caffeic Acid Is Non-Cytotoxic toward Experimental Cell Lines and Is Not a Substrate of P-gp

Before conducting further experiments, the cytotoxicity of caffeic acid was examined in HeLaS3, KB/VIN, Flp-In^TM^-293, and *ABCB1*/Flp-In^TM^-293 cell lines to select a rational concentration range. Caffeic acid exhibited higher than 80% cell viability in all tested cell lines under the treatment of 100 μg/mL for 72 h. Hence, the following assays were conducted with caffeic acid of not more than 100 μg/mL.

**Figure 2:**
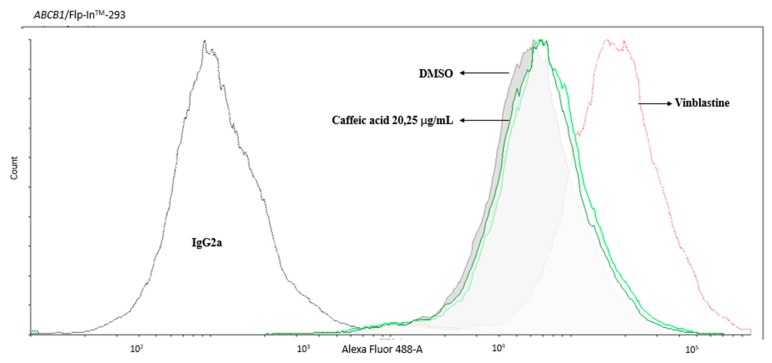
The result of MDR1 shift assay. The conformation of P-gp was not influenced under the treatment of 20 and 25 μg/mL caffeic acid. Vinblastine (a standard substrate of P-gp) was used as a positive control.

The first characteristic of caffeic acid on P-gp was demonstrated through MDR1 shift assay, which revealed whether a compound is a substrate of P-gp. P-gp’s substrates activate a conformational change detected by the structure-sensitive UIC2 antibody. As Figure 2 showed, the fluorescent peaks of caffeic acid 20 and 25 μg/mL did not shift to the right as the positive control vinblastine did, indicating that the conformation of P-gp was not influenced by caffeic acid. Therefore, caffeic acid is not P-gp’s substrate.

### 2.2. The Inhibitory Effects, Mechanisms and Binding Modes of Caffeic Acid on Human P-gp Function

**Figure 3:**
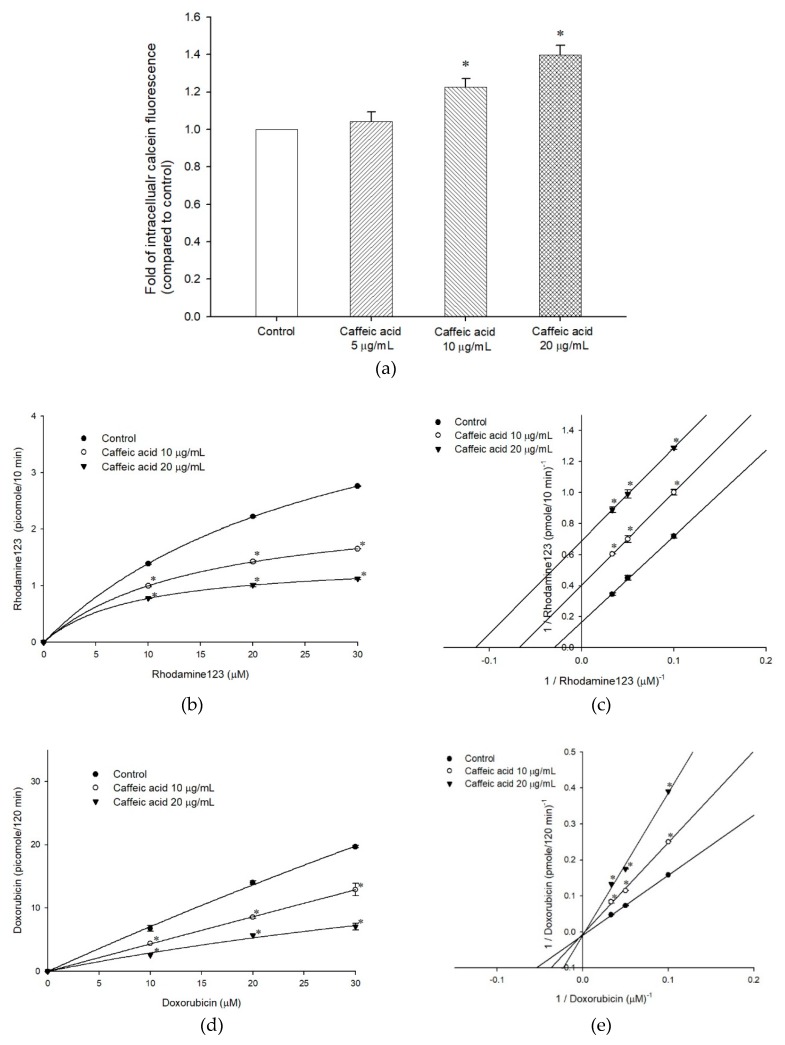

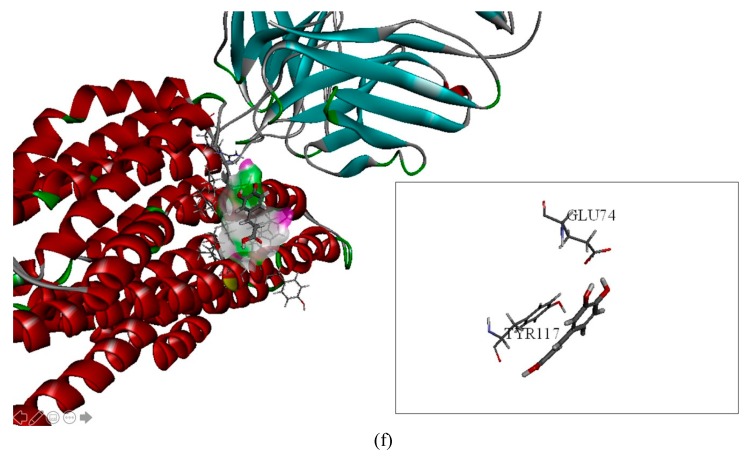
The effects of caffeic acid on human P-gp efflux function. (**a**) Intracellular calcein fluorescence with or without caffeic acid pretreatment in *ABCB1*/Flp-In^TM^-293 cell line (over-expressing human P-gp). (**b**) Michaelis-Menten kinetics of rhodamine123 efflux with or without caffeic acid pretreatment in *ABCB1*/Flp-In^TM^-293. (**c**) Lineweaver-Burk plot analyses of caffeic acid on the transport of rhodamine123 in human P-gp. (**d**) Michaelis-Menten kinetics of doxorubicin efflux with or without caffeic acid pretreatment in *ABCB1*/Flp-In^TM^-293. (**e**) Lineweaver-Burk plot analyses of caffeic acid on the transport of doxorubicin in human P-gp. * denotes *p* < 0.05 compared with the control group. Data were presented as mean ± SE of at least three experiments, each in triplicate. (**f**) Molecular docking analysis of caffeic acid (PubChem CID: 689043) docked posed of compounds in the P-gp (PDB entry 6QEX) binding pocket of 3D structure.

The inhibitory effect of caffeic acid on P-gp function was screened with calcein-AM accumulation assay. Calcein-AM is a non-florescent substance and P-gp’s substrate. It will be transformed to fluorescent calcein (not a P-gp substrate) by cell esterase. Therefore, under the treatment of P-gp’s inhibitor, the intracellular calcein fluorescence is higher than the normal condition. The results of caffeic acid are revealed in Figure 3a. When *ABCB1*/Flp-In^TM^-293 cell line was treated with caffeic acid in amounts of 5, 10, and 20 μg/mL, the intracellular calcein fluorescence was increased in a concentration-dependent manner. Hence, the efflux function of P-gp could be inhibited by caffeic acid.

Caffeic acid’s inhibitory effects and mechanisms were further demonstrated via the other two substrates of P-gp, rhodamine123 and doxorubicin. As Figure 3b showed, the efflux of fluorescent substrate rhodamine123 was inhibited by caffeic acid 10 and 20 μg/mL treatment and followed Michaelis-Menten kinetics. The Lineweaver-Burk plot (Figure 3c) indicated that caffeic acid inhibited rhodamine123 efflux in an uncompetitive pattern, both V_max_ and K_m_ decreased when the *ABCB1*/Flp-In^TM^-293 cell line was treated with increased caffeic acid concentrations (Table 1). Same as rhodamine123, the efflux of the chemotherapeutic drug doxorubicin was also inhibited by caffeic acid dose-dependently (Figure 3d). However, the inhibitory mechanism of caffeic acid on doxorubicin was competitive inhibition, different from rodamine123 (Figure 3e). When the *ABCB1*/Flp-In^TM^-293 cell line was treated with an increased concentration of caffeic acid, the K_m_ (affinity) increased accordingly and the V_max_ remained constant (Table 1).

In order to investigate the supposed binding pattern and possible interaction between the ligand of caffeic acid and pocket of P-gp, the ligand of caffeic acid was virtually docked to the crystal structures of the ligand-binding domain of P-gp using the docking program CDOCKER. The virtual binding result is shown in Figure 3f. The docking results showed that caffeic acid had the the best binding energies active site of P-gp with a -CDOCKER energy score of 20.1292, and binding energy was 44.4058 Kcal/mol. The binding model clearly indicated that the caffeic acid bound to P-gp with residues GLU74 and TRY117. Our docking results further demonstrated the binding behavior between P-gp and caffeic acid, providing insight into the design of novel P-gp modulators.

**Figure 4:**
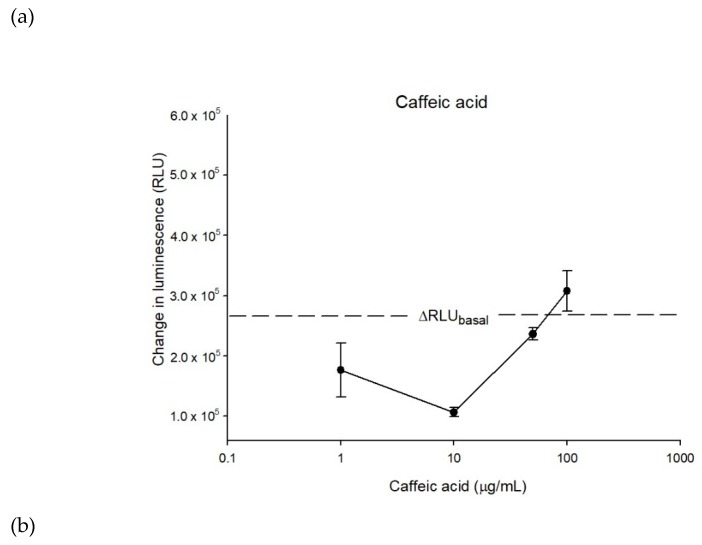

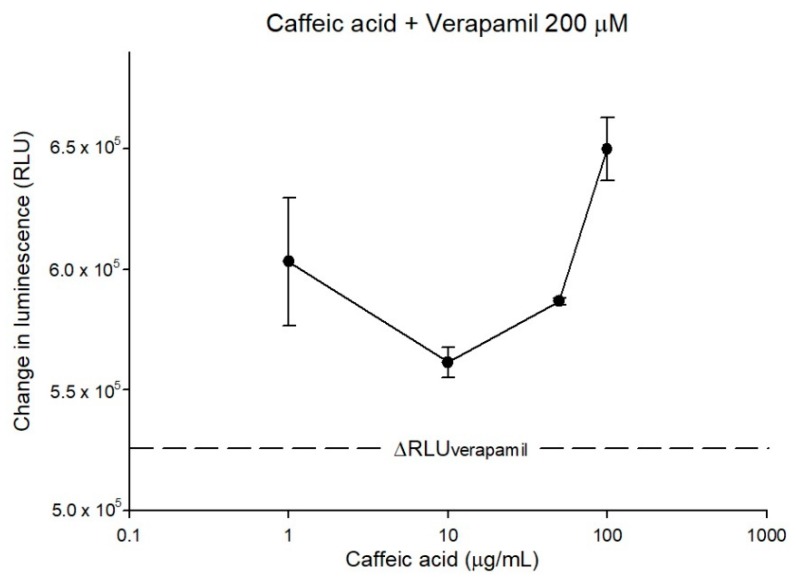
The P-gp ATPase modulating effects of caffeic acid. (**a**) Caffeic acid stimulated ATPase activity dose-dependently (10–100 μg/mL). (**b**) The verapamil-stimulated ATPase activity was further stimulated by caffeic acid. Data were analyzed in terms of the change of luminescence (ΔRLU). Data were presented as mean ± SE of at least three experiments, each in triplicate.

The interaction between caffeic acid and ATP binding site of P-gp was carried out with Pgp-Glo^TM^ Assay System. As Figure 4a shows, when the P-gp membrane was treated with caffeic acid with amounts of 1, 10, and 50 μg/mL, the basal P-gp ATPase activity was inhibited. On the other hand, the ATPase activity was stimulated under the treatment of 100 μg/mL caffeic acid. Besides, when combining caffeic acid with 200 μM verapamil, the elevated ATPase activity produced by verapamil was further stimulated and especially high under 100 μg/mL concentration (Figure 4b).

### 2.3. The Influences of Caffeic Acid on Human P-gp Expression

**Figure 5:**
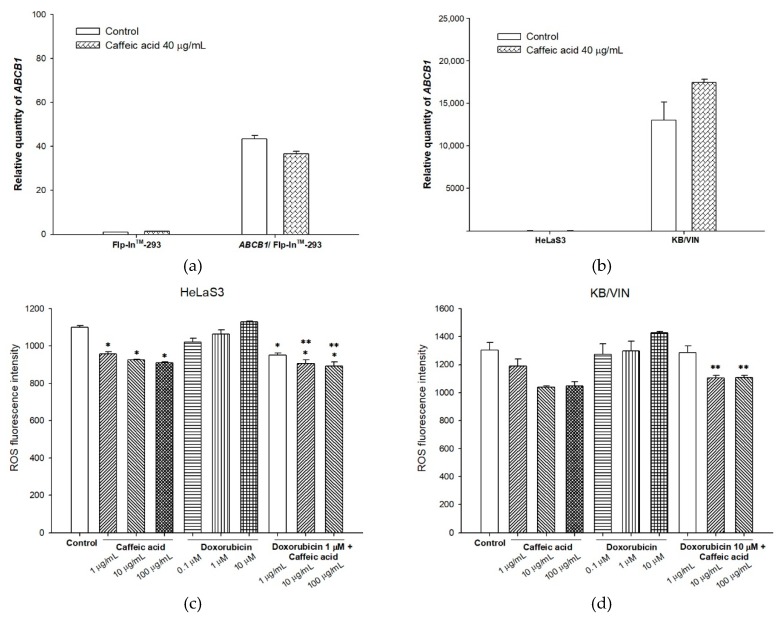
The modulating effects of caffeic acid on human P-gp expression. (**a**) The *ABCB1* expression in *ABCB1*/Flp-In^TM^-293 was slightly down-regulated after treating the cells with 40 μg/mL caffeic acid for 72 h. (**b**) The *ABCB1* expression in KB/VIN was slightly up-regulated after treating the cells with 40 μg/mL caffeic acid for 72 h. (**c**) The intracellular ROS production under the treatment of caffeic acid with or without doxorubicin in HeLaS3. (**d**) The intracellular ROS production under the treatment of caffeic acid with or without doxorubicin in KB/VIN. Data were presented as mean ± SE of at least three experiments, each in triplicate. * denotes *p* < 0.05 compared with the control group. ** denotes *p* < 0.05 compared with the doxorubicin 1 μM group in Figure 5c and doxorubicin 10 μM group in Figure 5d.

The modulatory ability of caffeic acid on *ABCB1* gene expression was performed in both *ABCB1*/Flp-In^TM^-293 and KB/VIN cell lines. In *ABCB1* overexpressing cell line *ABCB1*/Flp-In^TM^-293, caffeic acid after 72 h treatment slightly down-regulated the expression of P-gp (Figure 5a). Nevertheless, the same treatment for MDR cancer cell line KB/VIN exhibited the opposite phenomenon. Caffeic acid elevated *ABCB1* gene expression under 72 h treatment (Figure 5b).

To study whether the regulation of *ABCB1* gene expression was related to the intracellular reactive oxygen species status, the intracellular total ROS activity assay was performed. As Figure 5c,d indicates, caffeic acid significantly decreased ROS production in HeLaS3 cell line and slightly decreased ROS production in KB/VIN cell line. When both cell lines were treated with chemotherapeutic drug doxorubicin and caffeic acid, the ROS production exhibited no difference compared to caffeic acid treatment alone. However, the doxorubicin-induced oxidative challenge was significantly reversed by caffeic acid in amounts of 10 μg/mL and 100 μg/mL in both HeLaS3 and KB/VIN cell lines.

### 2.4. The Modulating Effects of Caffeic Acid on Cancer Multi-Drug Resistance

**Figure 6:**
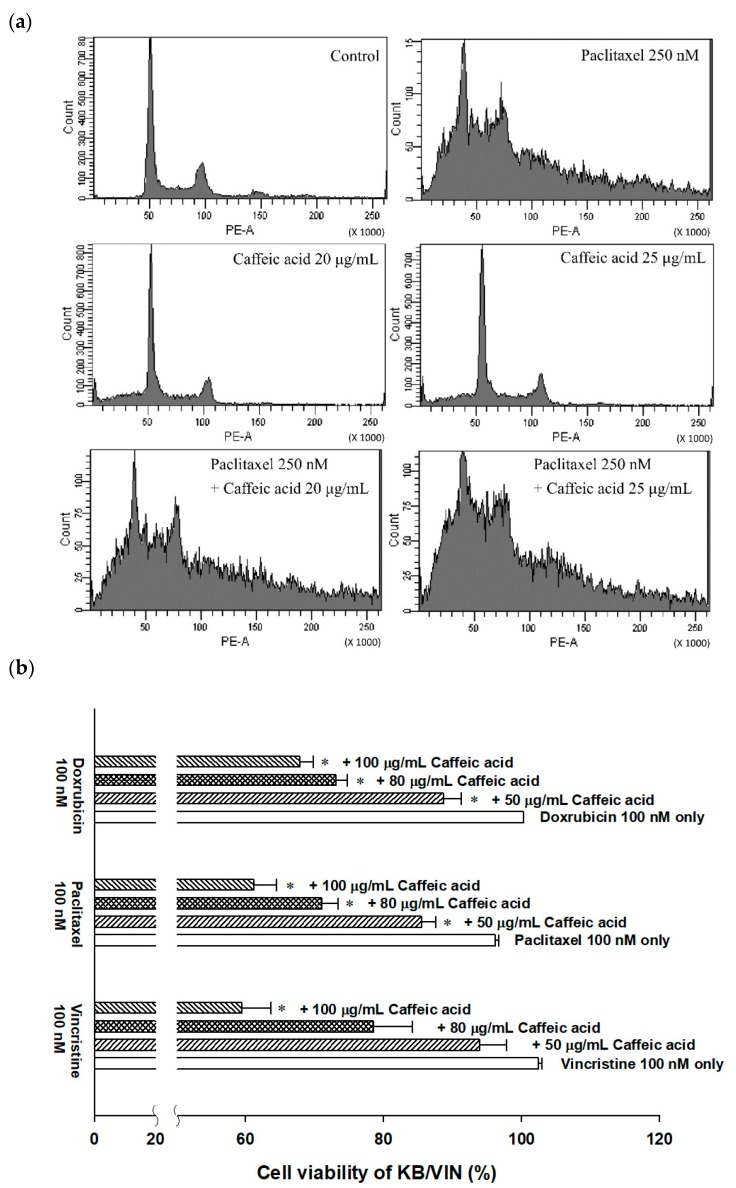

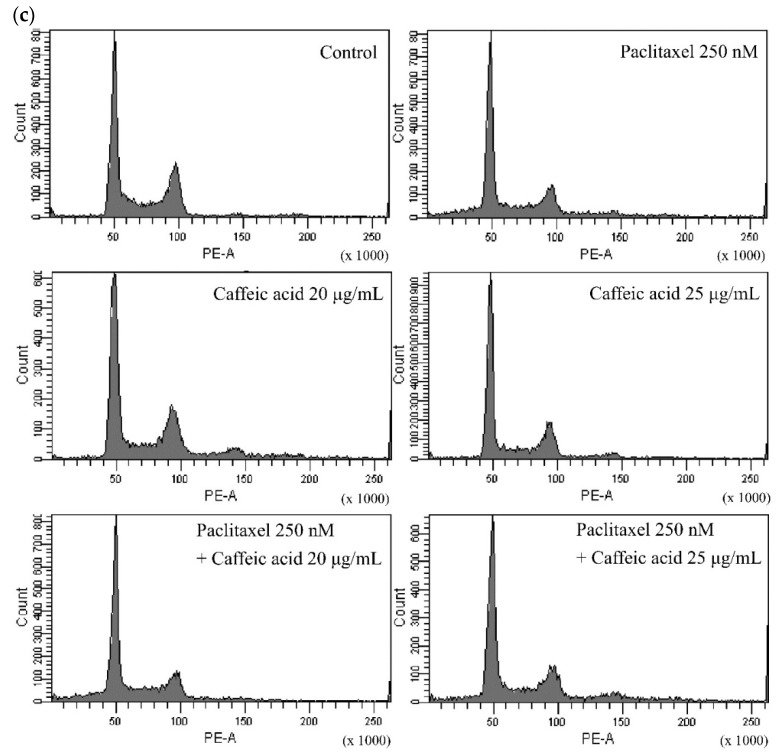
The cytotoxicity-enhancing effects of combinatorial treatment with caffeic acid. (**a**) The cell cycle distribution of 72 h treatment in *ABCB1*/Flp-In^TM^-293 cell line. (**b**) The cell viability of KB/VIN under the treatment of chemotherapeutic drugs with or without caffeic acid. Data were presented as mean ± SE of at least three experiments, each in triplicate. * *p* < 0.05 as compared to the chemotherapeutic drug treatment (doxorubicin, paclitaxel, or vincristine) without caffeic acid. (**c**) The cell cycle distribution of 72 h treatment in KB/VIN cell line.

The MDR reversal ability of caffeic acid was examined in both P-gp over-expressing cell line *ABCB1*/Flp-In^TM^-293 and MDR cancer cell line KB/VIN. As Table 2 shows, 30 μg/mL caffeic acid reversed vincristine, paclitaxel, and doxorubicin resistance by 3.90, 4.96, and 15.11-fold, respectively. The IC_50_ of doxorubicin decreased from 9023.61 nM to 569.90 nM with the treatment of caffeic acid 30 μg/mL in *ABCB1*/Flp-In^TM^-293. The MDR reversal phenomenon was further approved and analyzed by cell cycle. Compared to paclitaxel alone treatment, the addition of caffeic acid 20 and 25 μg/mL significantly increased subG1 population (from 11.3% to 24.1% and 33.0%), indicating that the cell underwent obvious apoptosis under combinatorial treatment (Figure 6a and Table 3).

In MDR cancer cell line KB/VIN, with 100 μg/mL caffeic acid treatment, the cytotoxicity of 100 nM doxorubicin, paclitaxel, and vincristine significantly increased. The cell viability decreased from nearly 100% to 67.91%, 61.18%, and 59.50% for doxorubicin, paclitaxel, and vincristine, respectively. In addition, the cytotoxic-enhancing ability of caffeic acid on chemotherapeutic drugs was in a dose-dependent manner (Figure 6b). However, the further cell cycle analyses showed that the combination of caffeic acid and paclitaxel did not prominently increase the apoptosis of KB/VIN cells, revealing distinct cell effects among *ABCB1*/Flp-In^TM^-293 and KB/VIN (Figure 6c and Table 3).

## 3. Discussion

Caffeic acid, a dietary non-flavonoid phenolic compound, has been a popular candidate among several research fields. The present study has demonstrated its usability in cancer MDR. Caffeic acid can attenuate this severe resistant problem by inhibiting the efflux function of human P-gp. Through diverse modulating mechanisms, caffeic acid helps resistant cancer cells retain chemotherapeutic drugs inside their cells, promoting further apoptosis and cell death.

Through investigating the history of P-gp inhibitor development, the ideal characteristics of potential candidates have been revealed. The inhibitor itself is not a substrate of P-gp, but is one of the favorable properties [19]. Our present research performed an experiment and the results indicated that caffeic acid was not P-gp’s substrate. In this way, more caffeic acid could stay inside the cells to help P-gp inhibition, resulting in a higher intracellular chemotherapeutic drugs concentration.

The inhibitory effects of caffeic acid on P-gp efflux function were demonstrated on three P-gp fluorescent substrates, calcein-AM, rhodamine123, and doxorubicin. The different binding modes of each substrate revealed the inhibitory mechanisms of caffeic acid on P-gp drug binding sites. A previous investigation found that doxorubicin was a R-site substrate while rhodamine123 exhibited both M and R sites binding affinity [37,38,39]. Our efflux assay results indicated that caffeic acid showed uncompetitive inhibition on rhodamine123 transport and competitive inhibition on doxorubicin transport. Therefore, caffeic acid might compete the R drug binding site with doxorubicin, resulting in efflux inhibition. In terms of rhodamine123 inhibition, caffeic acid exhibited an allosteric modulation on M site, indirectly prohibiting the pump out behavior of P-gp.

In addition to drug binding sites, the interaction between caffeic acid and ATP binding sites of P-gp was also studied. According to the tested compound’s behavior toward P-gp ATPase regulation, substances could be categorized into three classes: dual regulators, stimulators, and inhibitors [40,41]. Dual regulators stimulate both basal and verapamil-stimulated ATPase activity at a lower dose, but inhibit the activity at a higher dose, such as paclitaxel and vinblastine. Stimulators like valinomycin and bisantrene increase ATPase activity dose-dependently while inhibitors decrease both basal and verapamil-stimulated ATPase activity, such as rapamycin and cyclosporine A. The results of ATPase assay in the present study indicated that caffeic acid exhibited stimulatory activity from 10 μg/mL to 100 μg/mL in a dose-dependent manner. Therefore, caffeic acid was an ATPase stimulator. Besides, the results of verapamil-stimulated ATPase activity further revealed the binding behavior of caffeic acid on ATPase binding sites. Caffeic acid increased verapamil-stimulated ATPase activity regardless of the dose, implying its binding site on ATPase was different from verapamil. This allosteric stimulation advanced the consumption of ATP, indirectly inhibiting P-gp efflux function.

Whether the promising P-gp inhibitory effects of caffeic acid were helpful in reversing cancer MDR was than studied in our following experiments. In *ABCB1*/Flp-In^TM^-293 P-gp over-expressing cell line, caffeic acid significantly decreased the required doses of chemo-agents, including vincristine, paclitaxel, and doxorubicin. Under the treatment of 30 μg/mL caffeic acid, the IC_50_ of paclitaxel largely decreased from 604.09 nM to 121.55 nM. This advanced cytotoxicity was related to the increased apoptotic effects revealed by cell cycle assay results. With caffeic acid as a combinatory agent, the percentage of subG1 apoptotic population induced by paclitaxel significantly increased in a dose-dependent manner. The above results were consistent with previous researches, which revealed that caffeic acid could sensitize ovarian carcinoma cells and lung cancer cells to cisplatin and paclitaxel, respectively [33,42]. Caffeic acid exhibited chemo-sensitizing effects in the combination group by cell cycle arresting in G2/M (caffeic acid 20 μg/mL with paclitaxel) and G1 (caffeic acid 25 μg/mL with paclitaxel). These effects were not only due to the modulation of P-gp, other cellular targets and multiple mechanistic possibilities may be involved and need further investigation. The MDR reversing ability of caffeic acid was also investigated in MDR cancer cell line KB/VIN. The results exhibited a trend on increasing the cytotoxicity of chemo-agents. With the treatment of 100 μg/mL caffeic acid, the cell viability decreased from nearly 100% to 67.91%, 61.18%, and 59.50% for doxorubicin, paclitaxel, and vincristine, respectively. However, compared to the promising results in *ABCB1*/Flp-In^TM^-293 cell line, the MDR modulating effects of caffeic acid in KB/VIN seemed to be less potent and did not show increased apoptosis in the cell cycle analyses, exhibiting cell type-dependent effects. This phenomenon could be explained by the regulation of caffeic acid on P-gp expression. As Figure 5a,b shows, caffeic acid slightly decreased *ABCB1* gene expression in *ABCB1*/Flp-In^TM^-293 but increased the expression level in KB/VIN cell line. This up-regulating trend in KB/VIN diminished the functional inhibitory potency of caffeic acid, resulting in weaker MDR reversing effects. Previous research has revealed that the oxidative stress might have a role in the regulation of P-gp expression [43]. As caffeic acid exhibited significant ROS-related anti-oxidant effects, the influence of caffeic acid on ROS production in KB/VIN cell line was performed. The results showed that caffeic acid significantly decreased ROS production in HeLaS3 cell line and slightly decreased ROS production in KB/VIN cell line. However, the doxorubicin-induced oxidative challenge was significantly reversed by caffeic acid in amounts of 10 μg/mL and 100 μg/mL in both HeLaS3 and KB/VIN cell lines. Therefore, the relationship between the reactive oxygen species levels and the up-regulation of *ABCB1* gene in KB/VIN might be related to the insufficient ROS regulation of caffeic acid. The above results indicated that the MDR reversal effects of caffeic acid might be cell line-dependent and warrant further detailed investigation.

The present study provided in-depth and comprehensive researches on the relationship between caffeic acid and human P-gp, and demonstrated the ability of caffeic acid on sensitizing MDR cancer cells toward chemotherapeutic drugs treatment. In order for caffeic acid to find a role in clinical application, some attempts could be applied to this phenolic prototype agent, including structural modification and pharmaceutical design.

## 4. Materials and Methods

### 4.1. Chemicals and Reagents

Acetic acid, β-Mercaptoethanol (β-ME), caffeic acid, dimethyl sulfoxide (DMSO), ethanol (Absolute; analytical grade), paclitaxel, rhodamine 123, sulforhodamine B (SRB), trichloroacetic acid (TCA), tris base, (±)-verapamil, and vincristine were obtained from Sigma-Aldrich Co. (St Louis, MO, USA). Calcein-AM was from AAT Bioquest (Sunnyvale, CA, USA), and doxorubicin was from US Biological (Woburn, MA, USA). Dulbecco’s Modified Eagle Medium, RPMI 1640 medium, fetal bovine serum (FBS), phosphate buffered saline (PBS; pH 7.2), Trypsin-EDTA, and hygromycin B were purchased from Invitrogen (Carlsbad, CA, USA). Zeocin was from InvivoGen (San Diego, CA, USA).

### 4.2. Cell Lines

Human cervical epithelioid carcinoma HeLaS3 was purchased from Bioresource Collection and Research Center (Hsinchu, Taiwan), and the multi-drug resistant human cervical cancer cell line KB/VIN was kindly provided by Dr. Kuo-Hsiung Lee (University of North Carolina, Chapel Hill, NC, USA) and maintained with vincristine regularly. The human P-gp stable expression cells (*ABCB1*/Flp-In^TM^-293) and parental cell line Flp-In^TM^-293 were constructed as previously described [44]. All cells were cultured in DMEM or RPMI-1640 containing 10% FBS at 37 °C in a humidified atmosphere of 5% CO_2_.

### 4.3. Cytotoxicity Determination Assay (SRB Assay)

The method has been described in our previous research [45]. Briefly, after 72 h of treatment of a series of concentrations of chemotherapeutic drugs with or without caffeic acid, 50% trichloroacetic acid (TCA) was added to fix cells for 30 min, and then the cells were washed with water and air-dried. After that, cells were stained with 0.04% sulforhodamine B (SRB) for 30 min, and then the unbound dye was removed by washing cells with 1% acetic acid and air-dried. The bound stain was solubilized in 10 mM Tris Base and the absorbance was measured using a BioTek Synergy HT Multi-Mode Microplate Reader (Winooski, VT, USA) at 515 nm.

### 4.4. MDR1 Shift Assay

The method has been described in our previous research [46]. The conformation change of P-gp after the addition of caffeic acid was examined by using a MDR1 Shift Assay kit (EMD Millipore Corp., Billerica, MA, USA) according to the manufacturer’s protocol. UIC2 shift was shown in the presence of a P-gp substrate such as vinblastine. A total of 5 × 10^5^–1 × 10^6^ cells were prepared per reaction and resuspended with warm UIC2 binding buffer. Cells were incubated at 37 °C for 10 min and then treated with DMSO or vinblastine or test compounds. Cells were incubated at 37 °C for 30 min and then treated with IgG2a (negative control antibody) or UIC2 working solution (P-gp conformational sensitive antibody). Cells were incubated at 37 °C for 15 min and then washed with iced UIC2 binding buffer twice. A secondary antibody, goat anti-mouse IgG ALEXA 488, was added at 4 °C for 15 min, and then iced UIC2 binding buffer was added. The fluorescence was measured by FACS analysis (BD FACSCanto^TM^ II System, South City-I, Haryana, India).

### 4.5. Intracellular Calcein Accumulation Assay

The method has been described in our previous research [46]. For the screening of an inhibitory effect on human P-gp efflux function, intracellular calcein accumulation assay was performed. Briefly, 1 × 10^5^ cells/well were seeded in 96-well black plates for 24 h. Before the assay, cells were washed and pre-incubated with warm Hanks’ balanced salt solution (HBSS) for 30 min and subsequently with caffeic acid for 30 min. After pre-incubation and three times washing with PBS, the calcein-AM was added (substrate of P-gp), and the calcein fluorescence generated within the cells was detected by BioTek Synergy HT Multi-Mode Microplate Reader using an excitation wavelength of 485 nm and emission wavelength of 528 nm at 37 °C temperature every 3 min for 30 min. Each experiment was performed at least three times, each in triplicate on different days.

### 4.6. Rhodamine123 and Doxorubicin Efflux Assay

The method has been described in our previous research [46]. 1 × 10^5^ cells/well were placed on 96-well plates and incubated overnight. Before the efflux assay, cells were washed and pre-incubated with warm HBSS for 30 min, and subsequently with caffeic acid for 30 min. Then, the cells were treated with rhodamine123 for 30 min or doxorubicin for 3 h at 37 °C. After being washed with warm PBS, cells were allowed to efflux fluorescent rhodamin123 and doxorubicin for 10 min and 2 h, respectively. Supernatant samples (100 μL) were transferred to 96-well black plates. The fluorescence of rhodamine123 and doxorubicin was measured using a BioTek Synergy HT Multi-Mode Microplate Reader (excitation/emission: 485/528 nm for rhodamine123, 485/590 nm for doxorubicin). Each experiment was performed at least three times, each in triplicate on different days. Kinetic parameters were estimated by nonlinear regression using Scientist v2.01 (MicroMath Scientific Software, Salt Lake City, UT, USA) according to the following equation:

$$V=\frac{V_{\max}\;\times \;C}{K_{m}\;+\;C}$$

where V denoted the efflux rate; V_max_, the maximal efflux rate; K_m_, the Michaelis-Menten constant; and C is the substrate concentration.

### 4.7. P-gp ATPase Activity Assay

The method has been described in our previous research [46]. For the evaluation of P-gp ATPase activity of caffeic acid, Pgp-Glo^TM^ Assay System from Promega (Madison, WI, USA) was used. In a 96-well untreated white plate, 25 μg of recombinant human P-gp membranes were incubated with Pgp-Glo^TM^ Assay Buffer (untreated control), 200 μM verapamil (positive control for drug induced P-gp ATPase activity), 100 μM sodium orthovanadate (selective inhibitor for P-gp ATPase activity), or a series of concentrations of caffeic acid. The reaction was initiated by adding 5 mM MgATP and incubated for 40 min at 37 °C, followed by stopping the reaction with 50 μL ATPase Detection Reagent for 20 min at room temperature. Luminescence was measured using a BioTek Synergy HT Multi-Mode Microplate Reader, and data were presented as Change in Luminescence (ΔRLU).

### 4.8. Real-Time Quantitative RT-PCR

The method has been described in our previous research [46]. *ABCB1* mRNA expression levels were quantified by real-time RT-PCR. Total RNA was extracted from HeLaS3, KB/VIN, Flp-In^TM^-293, and *ABCB1*/Flp-In^TM^-293 cells using Qiagen RNeasy kit (Valencia, CA, USA). Taqman Assay-On-Demand^TM^ reagents of primers and probes for *ABCB1* (Hs00184500_m1) and *GAPDH* (Hs02758991_g1) genes were provided by Applied Biosystem (Foster City, CA, USA). The relative *ABCB1* mRNA expression levels were normalized to the amount of *GAPDH* in the same cDNA and evaluated by StepOnePlus^TM^ Real-Time PCR System (Applied Biosystems^®^).

### 4.9. Intracellular Total ROS Activity Assay

The influence of caffeic acid on intracellular reactive oxygen species (ROS) was evaluated with Cell Meter™ Fluorimetric Intracellular Total ROS Activity Assay Kit (Catalog number: 22900) purchased from AAT Bioquest (Sunnyvale, CA, USA). Briefly, 4 × 10^4^ cells/well were seeded in 96-well black plates for 24 h. Then the cells were stained with Amplite^TM^ ROS Green working solution for 1 h; after that, caffeic acid with or without chemotherapeutic drugs were added to induce ROS production at room temperature for at least 15 min. The fluorescence was measured using a BioTek Synergy HT Multi-Mode Microplate Reader at 490/525 nm (same as FITC filter).

### 4.10. Cell Cycle Analysis

The method has been described in our previous research [45]. Cells were plated to 6-well plates with serum-free medium for starvation. Twenty-four hours later, cells were treated with chemotherapeutic drugs with or without caffeic acid for 72 h. After that, cells were harvested and washed in cold phosphate-buffered saline (PBS), followed by fixing in ice-cold 70% ethanol for at least 24 h. Then, cells were incubated with 50 μg/mL PI at 4 °C for 24 h in the dark. Cells were then analyzed by FACS analysis (BD FACSCanto^TM^ II System with excitation laser 488 nm, measuring at emission 575 nm for PI).

### 4.11. Molecular Docking

Molecular docking helps us in predicting the intermolecular framework formed between a protein and a small molecule and suggests the binding modes responsible for inhibition of the protein. In this study, the existing structure of P-gp (PDB entry 6QEX) was used as a template for docking caffeic acid (PubChem CID: 689043) putative ligands using Discovery Studio 4.5. After removing all crystallized H_2_O molecules from the former construction, hydrogen was added into the CDOCKER module. CDOCKER is a powerful CHARMm-based docking method that has been used to generate highly accurate docked poses. In this refinement application, the ligands were conceded to tilt around the rigid receptor [47].

### 4.12. Statistical Analysis

Statistical differences were evaluated by ANOVA followed by post hoc analysis (Tukey’s test) or Student’s t-test. The statistical significance was set at *p* value < 0.05.

## Figures and Tables

**Table 1:** The effects of caffeic acid on the transport of rhodamine123 and doxorubicin by human P-gp.

	Nonlinear Kinetic Parameters		
*ABCB1*/Flp-In^TM^-293	V_m_ (pmole/10 min)	K_m_ (µM)	
Rhodamine123 only	9.04 ± 1.00	56.52 ± 6.97	
+ Caffeic acid, 10 µg/mL	2.47 ± 0.03*	15.93 ± 0.18 *	
+ Caffeic acid, 20 µg/mL	1.57 ± 0.04 *	10.72 ± 0.30 *	
K_i_			253.44 ± 2.64
*ABCB1*/Flp-In^TM^-293	V_m_ (pmole/120 min)	K_m_ (µM)	
Doxorubicin only	107.52 ± 0.001	179.81 ± 0.001	
+ Caffeic acid, 10 µg/mL	108.31 ± 0.68	234.76 ± 1.52 *	
+ Caffeic acid, 20 µg/mL	107.52 ± 0.001	426.16 ± 0.00 *	
K_i_			14.13 ± 0.005

* *p* < 0.05 as compared to the rhodamine123 or doxorubicin transport without caffeic acid.

**Table 2:** The reversal effects of caffeic acid on chemotherapeutic drug resistance in P-gp over-expressing cell line *ABCB1*/Flp-In^TM^293.

Cell Line	Flp-In^TM^293		*ABCB1*/Flp-In^TM^293	
Compound	IC_50_ ± S.E. (nM)	RF	IC_50_ ± S.E. (nM)	RF
Vincristine	9.34 ± 0.43	1.00	778.11 ± 14.77	1.00
+ 30 μg/mL Caffeic acid	3.37 ± 4.30	2.77	198.04 ± 6.62	3.90
+ 20 μg/mL Caffeic acid	7.08 ± 0.09	1.31	557.46 ± 8.70	1.40
+ 10 μg/mL Caffeic acid	9.11 ± 0.32	1.02	615.03 ± 3.09	2.26
Paclitaxel	89.99 ± 0.50	1.00	604.09 ± 7.09	1.00
+ 30 μg/mL Caffeic acid	40.9 ± 0.50	2.20	121.55 ± 13.50	4.96 *
+ 20 μg/mL Caffeic acid	79.3 ± 0.67	1.13	313.06 ± 37.71	1.92
+ 10 μg/mL Caffeic acid	86.9 ± 0.12	1.03	597.87 ± 11.25	1.01
Doxorubicin	8.55 ± 0.19	1.00	9023.61 ± 272.90	1.00
+ 30 μg/mL Caffeic acid	4.07 ± 4.49	2.10	596.90 ± 24.18	15.11 *
+ 20 μg/mL Caffeic acid	7.34 ± 4.67	1.20	1299.7 ± 37.18	6.94 *
+ 10 μg/mL Caffeic acid	8.48 ± 2.58	1.00	2628.1 ± 24.49	3.43

* *p* < 0.05 as compared to the chemotherapeutic drug treatment (vincristine, paclitaxel, or doxorubicin) without caffeic acid. The reversal fold (RF) was calculated by dividing the individual IC_50_ of chemotherapeutic drugs by the IC_50_ of chemotherapeutic drugs in the presence of caffeic acid.

**Table 3:** The percentage of each cell cycle phase under various treatments in *ABCB1*/Flp-In^TM^-293 cell line and KB/VIN cell line.

***ABCB1*/Flp-In^TM^-293**	**Percentage of Phase ± SE (%)**			
	**Sub G1**	**G0/G1**	**S**	**G2/M**
Control	0.4 ± 0.17	35.7 ± 1.5	46.2 ± 2.9	17.6 ± 4.3
Paclitaxel 250 nM	11.3 ± 0.2	27.4 ± 0.6	29.3 ± 1.6	31.8 ± 1.3
Caffeic acid 20 μg/mL	3.7 ± 0.6	27.4 ± 0.6	29.3 ± 1.6	31.8 ± 1.3
Caffeic acid 25 μg/mL	1.2 ± 0.1	41.7 ± 0.5	37.7 ± 0.2	19.2 ± 0.6
Paclitaxel 250 nM + Caffeic acid 20 μg/mL	24.1 ± 0.3	36.6 ± 1.4	24.1 ± 1.8	15.0 ± 0.5
Paclitaxel 250 nM + Caffeic acid 25 μg/mL	33.0 ± 9.0	27.5 ± 8.5	29.7 ± 4.8	14.8 ± 0.6
**KB/VIN**	**Percentage of Phase ± SE (%)**			
	**Sub G1**	**G0/G1**	**S**	**G2/M**
Control	0.6 ± 0.07	37.3 ± 4.0	39.2 ± 0.7	22.9 ± 3.3
Paclitaxel 250 nM	12.8 ± 1.5	44.6 ± 1.0	29.2 ± 0.4	13.3 ± 0.1
Caffeic acid 20 μg/mL	1.5 ± 0.04	40.3 ± 0.9	45.5 ± 1.3	12.8 ± 0.5
Caffeic acid 25 μg/mL	1.3 ± 0.1	37.2 ± 0.4	50.1 ± 0.5	11.4 ± 0.3
Paclitaxel 250 nM + Caffeic acid 20 μg/mL	13.2 ± 1.2	44.3 ± 0.5	31.6 ± 1.0	11.0 ± 0.2
Paclitaxel 250 nM + Caffeic acid 25 μg/mL	12.7 ± 1.3	46.9 ± 0.1	23.6 ± 1.5	16.8 ± 0.2

## References

[1] Silva R., Vilas-Boas V., Carmo H., Dinis-Oliveira R.J., Carvalho F., de Lourdes Bastos M., Remiao F. (2015). Modulation of P-glycoprotein efflux pump: Induction and activation as a therapeutic strategy. Pharmacol. Ther..

[2] Kumar A., Jaitak V. (2019). Natural products as multidrug resistance modulators in cancer. Eur. J. Med. Chem..

[3] Kathawala R.J., Wang Y.J., Ashby C.R., Chen Z.S. (2014). Recent advances regarding the role of ABC subfamily C member 10 (ABCC10) in the efflux of antitumor drugs. Chin. J. Cancer.

[4] Shi R., Wang C., Fu N., Liu L., Zhu D., Wei Z., Zhang H., Xing J., Wang Y. (2019). Downregulation of cytokeratin 18 enhances BCRP-mediated multidrug resistance through induction of epithelial-mesenchymal transition and predicts poor prognosis in breast cancer. Oncol. Rep..

[5] Das M., Law S. (2018). Role of tumor microenvironment in cancer stem cell chemoresistance and recurrence. Int. J. Biochem. Cell Biol..

[6] Giacomini K.M., Huang S.M., Tweedie D.J., Benet L.Z., Brouwer K.L., Chu X., Dahlin A., Evers R., Fischer V., Hillgren K.M. (2010). Membrane transporters in drug development. Nat. Rev. Drug Discov..

[7] Uddin M.B., Roy K.R., Hosain S.B., Khiste S.K., Hill R.A., Jois S.D., Zhao Y., Tackett A.J., Liu Y.Y. (2019). An *N*(6)-methyladenosine at the transited codon 273 of p53 pre-mRNA promotes the expression of R273H mutant protein and drug resistance of cancer cells. Biochem. Pharmacol..

[8] Bedi A., Barber J.P., Bedi G.C., el-Deiry W.S., Sidransky D., Vala M.S., Akhtar A.J., Hilton J., Jones R.J. (1995). BCR-ABL-mediated inhibition of apoptosis with delay of G2/M transition after DNA damage: A mechanism of resistance to multiple anticancer agents. Blood.

[9] Wilson C.S., Medeiros L.J., Lai R., Butch A.W., McCourty A., Kelly K., Brynes R.K. (2001). DNA topoisomerase IIalpha in multiple myeloma: A marker of cell proliferation and not drug resistance. Mod. Pathol..

[10] Filomeni G., Turella P., Dupuis M.L., Forini O., Ciriolo M.R., Cianfriglia M., Pezzola S., Federici G., Caccuri A.M. (2008). 6-(7-Nitro-2,1,3-benzoxadiazol-4-ylthio)hexanol, a specific glutathione S-transferase inhibitor, overcomes the multidrug resistance (MDR)-associated protein 1-mediated MDR in small cell lung cancer. Mol. Cancer Ther..

[11] Rodriguez-Antona C., Ingelman-Sundberg M. (2006). Cytochrome P450 pharmacogenetics and cancer. Oncogene.

[12] Shoemaker R.H. (2000). Genetic and epigenetic factors in anticancer drug resistance. J. Natl. Cancer Inst..

[13] Li H., Yang B.B. (2013). Friend or foe: The role of microRNA in chemotherapy resistance. Acta Pharm. Sin.

[14] Camidge D.R., Pao W., Sequist L.V. (2014). Acquired resistance to TKIs in solid tumours: Learning from lung cancer. Nat. Rev. Clin. Oncol..

[15] Paskeviciute M., Petrikaite V. (2019). Overcoming transporter-mediated multidrug resistance in cancer: Failures and achievements of the last decades. Drug Deliv. Transl. Res..

[16] Yakusheva E.N., Titov D.S. (2018). Structure and Function of Multidrug Resistance Protein 1. Biochem. Biokhimiia.

[17] Ling V., Thompson L.H. (1974). Reduced permeability in CHO cells as a mechanism of resistance to colchicine. J. Cell. Physiol..

[18] Mollazadeh S., Sahebkar A., Hadizadeh F., Behravan J., Arabzadeh S. (2018). Structural and functional aspects of P-glycoprotein and its inhibitors. Life Sci..

[19] Leopoldo M., Nardulli P., Contino M., Leonetti F., Luurtsema G., Colabufo N.A. (2019). An updated patent review on P-glycoprotein inhibitors (2011-2018). Expert Opin. Ther. Pat..

[20] Joshi P., Vishwakarma R.A., Bharate S.B. (2017). Natural alkaloids as P-gp inhibitors for multidrug resistance reversal in cancer. Eur. J. Med. Chem..

[21] O’Brien M.M., Lacayo N.J., Lum B.L., Kshirsagar S., Buck S., Ravindranath Y., Bernstein M., Weinstein H., Chang M.N., Arceci R.J. (2010). Phase I study of valspodar (PSC-833) with mitoxantrone and etoposide in refractory and relapsed pediatric acute leukemia: A report from the Children’s Oncology Group. Pediatr. Blood Cancer.

[22] Li W., Zhang H., Assaraf Y.G., Zhao K., Xu X., Xie J., Yang D.H., Chen Z.S. (2016). Overcoming ABC transporter-mediated multidrug resistance: Molecular mechanisms and novel therapeutic drug strategies. Drug Resist. Updat..

[23] Cripe L.D., Uno H., Paietta E.M., Litzow M.R., Ketterling R.P., Bennett J.M., Rowe J.M., Lazarus H.M., Luger S., Tallman M.S. (2010). Zosuquidar, a novel modulator of P-glycoprotein, does not improve the outcome of older patients with newly diagnosed acute myeloid leukemia: A randomized, placebo-controlled trial of the Eastern Cooperative Oncology Group 3999. Blood.

[24] Dash R.P., Jayachandra Babu R., Srinivas N.R. (2017). Therapeutic Potential and Utility of Elacridar with Respect to P-glycoprotein Inhibition: An Insight from the Published In Vitro, Preclinical and Clinical Studies. Eur. J. Drug Metab. Pharmacokinet..

[25] Han R.M., Zhang J.P., Skibsted L.H. (2012). Reaction dynamics of flavonoids and carotenoids as antioxidants. Molecules.

[26] Kumar S., Pandey A.K. (2013). Chemistry and biological activities of flavonoids: An overview. Sci. World J..

[27] Meyer A.S., Heinonen M., Frankel E.N. (1998). Antioxidant interactions of catechin, cyanidin, caffeic acid, quercetin, and ellagic acid on human LDL oxidation. Food Chem..

[28] Jaman M.S., Sayeed M.A. (2018). Ellagic acid, sulforaphane, and ursolic acid in the prevention and therapy of breast cancer: Current evidence and future perspectives. Breast Cancer (TokyoJpn.).

[29] Xing Y., Peng H.Y., Zhang M.X., Li X., Zeng W.W., Yang X.E. (2012). Caffeic acid product from the highly copper-tolerant plant Elsholtzia splendens post-phytoremediation: Its extraction, purification, and identification. J. Zhejiang Univ. Sci. B.

[30] Ahmed N., Escalona R., Leung D., Chan E., Kannourakis G. (2018). Tumour microenvironment and metabolic plasticity in cancer and cancer stem cells: Perspectives on metabolic and immune regulatory signatures in chemoresistant ovarian cancer stem cells. Semin. Cancer Biol..

[31] Khan F.A., Maalik A., Murtaza G. (2016). Inhibitory mechanism against oxidative stress of caffeic acid. J. Food Drug Anal..

[32] Ahn C.H., Choi W.C., Kong J.Y. (1997). Chemosensitizing activity of caffeic acid in multidrug-resistant MCF-7/Dox human breast carcinoma cells. Anticancer Res..

[33] Lin C.L., Chen R.F., Chen J.Y., Chu Y.C., Wang H.M., Chou H.L., Chang W.C., Fong Y., Chang W.T., Wu C.Y. (2012). Protective effect of caffeic acid on paclitaxel induced anti-proliferation and apoptosis of lung cancer cells involves NF-kappaB pathway. Int. J. Mol. Sci..

[34] Wu J., Omene C., Karkoszka J., Bosland M., Eckard J., Klein C.B., Frenkel K. (2011). Caffeic acid phenethyl ester (CAPE), derived from a honeybee product propolis, exhibits a diversity of anti-tumor effects in pre-clinical models of human breast cancer. Cancer Lett..

[35] Nabekura T., Kawasaki T., Furuta M., Kaneko T., Uwai Y. (2018). Effects of Natural Polyphenols on the Expression of Drug Efflux Transporter P-Glycoprotein in Human Intestinal Cells. Acs Omega.

[36] Takara K., Fujita M., Matsubara M., Minegaki T., Kitada N., Ohnishi N., Yokoyama T. (2007). Effects of propolis extract on sensitivity to chemotherapeutic agents in HeLa and resistant sublines. Phytother. Res. Ptr..

[37] Montanari F., Ecker G.F. (2015). Prediction of drug-ABC-transporter interaction--Recent advances and future challenges. Adv. Drug Deliv. Rev..

[38] Ferreira R.J., Ferreira M.J., dos Santos D.J. (2013). Molecular docking characterizes substrate-binding sites and efflux modulation mechanisms within P-glycoprotein. J. Chem. Inf. Model..

[39] Martinez L., Arnaud O., Henin E., Tao H., Chaptal V., Doshi R., Andrieu T., Dussurgey S., Tod M., Di Pietro A. (2014). Understanding polyspecificity within the substrate-binding cavity of the human multidrug resistance P-glycoprotein. FEBS J..

[40] Ambudkar S.V., Dey S., Hrycyna C.A., Ramachandra M., Pastan I., Gottesman M.M. (1999). Biochemical, cellular, and pharmacological aspects of the multidrug transporter. Ann. Rev. Pharmacol. Toxicol..

[41] Dey S., Ramachandra M., Pastan I., Gottesman M.M., Ambudkar S.V. (1997). Evidence for two nonidentical drug-interaction sites in the human P-glycoprotein. Proc. Natl. Acad. Sci. USA.

[42] Sirota R., Gibson D., Kohen R. (2017). The timing of caffeic acid treatment with cisplatin determines sensitization or resistance of ovarian carcinoma cell lines. Redox Biol..

[43] Huang C., Huang S., Li H., Li X., Li B., Zhong L., Wang J., Zou M., He X., Zheng H. (2018). The effects of ultrasound exposure on P-glycoprotein-mediated multidrug resistance in vitro and in vivo. J. Exp. Clin. Cancer Res..

[44] Sheu M.J., Teng Y.N., Chen Y.Y., Hung C.C. (2014). The functional influences of common ABCB1 genetic variants on the inhibition of P-glycoprotein by Antrodia cinnamomea extracts. PLoS ONE.

[45] Teng Y.-N., Wang Y.-H., Wu T.-S., Hung H.-Y., Hung C.-C. (2019). Zhankuic Acids A, B and C from Taiwanofungus camphoratus Act as Cytotoxicity Enhancers by Regulating P-Glycoprotein in Multi-Drug Resistant Cancer Cells. Biomolecules.

[46] Teng Y.-N., Hsieh Y.-W., Hung C.-C., Lin H.-Y. (2015). Demethoxycurcumin Modulates Human P-Glycoprotein Function via Uncompetitive Inhibition of ATPase Hydrolysis Activity. J. Agric. Food Chem..

[47] Wu G., Robertson D.H., Brooks C.L., Vieth M. (2003). Detailed analysis of grid-based molecular docking: A case study of CDOCKER-A CHARMm-based MD docking algorithm. J. Comput. Chem..

